# Performance and Hypothetical Impact on Joint Infection Management of the BioFire Joint Infection Panel: a Retrospective Analysis

**DOI:** 10.1128/jcm.00592-23

**Published:** 2023-07-13

**Authors:** Benjamin Berinson, Laura Spenke, Lukas Krivec, Konstantin Tanida, Anna Both, Johannes Keller, Tim Rolvien, Martin Christner, Marc Lütgehetmann, Martin Aepfelbacher, Till Orla Klatte, Holger Rohde

**Affiliations:** a Institute for Medical Microbiology, Virology and Hygiene, University Medical Center Hamburg-Eppendorf, Hamburg, Germany; b Department of Trauma and Orthopaedic Surgery, University Medical Center Hamburg-Eppendorf, Hamburg, Germany; Johns Hopkins University

**Keywords:** joint infection, synovial fluid, diagnostics, multiplex PCR, FilmArray, species identification, resistance, BioFire, diagnostic stewardship, joint infections, syndromic panel PCR

## Abstract

Pathogen identification is key in septic arthritis. Culture-based techniques are challenging, especially when patients have been pretreated with antibiotics or when difficult-to-culture bacteria are encountered. The BioFire joint infection assay (BJA) is a multiplex PCR panel which detects 31 of the most prevalent bacterial and fungal pathogens causing septic arthritis. Here, 123 cryoconserved contemporary synovial fluid samples from 120 patients underwent BJA analysis. Results were compared to those of culture-based diagnostics (standard of care [SOC]). Clinical data were collected, and the possible impact of the molecular diagnostic application on patient management was evaluated. Fifteen of 123 synovial fluid cultures grew bacterial pathogens. All on-panel pathogens (9/15) were correctly identified by the BJA. The BJA identified four additional bacterial pathogens in four SOC-negative cases. BJA sensitivity and specificity were 100% (95% confidence interval [CI], 69.2% to 100%) and 100% (95% CI, 96.8% to 100%), respectively. Compared to the SOC, the BJA would have resulted in faster provision of species identification and molecular susceptibility data by 49 h and 99 h, respectively. Clinical data analysis indicates that in BJA-positive cases, faster species ID could have led to timelier optimization of antibiotic therapy. This retrospective study demonstrates high sensitivity and specificity of the BJA to detect on-panel organisms in bacterial arthritis. The usefulness of the BJA in prosthetic-joint infections is limited, as important pathogens (i.e., coagulase negative staphylococci and Cutibacterium acnes) are not covered. Evidence from patient data analysis suggests that the assay might prove valuable for optimizing patient management in acute arthritis related to fastidious organisms or for patients who received antibiotics prior to specimen collection.

## INTRODUCTION

Joint infections are a serious and possibly life-threatening diseases. In Europe, the incidence of native joint arthritis ranges from 4 to 10 per 100,000 patient-years (1–3). Individuals at highest risk are young children and older adults (4). In addition to native-joint infections (NJI), infections associated with prosthetic joints are of ever-increasing importance. Likely related to the growing numbers of implanted prosthetic joints and a more senescent population (5), a recent report documented incidences ranging between 70 (females) and 180 (males) per 100,000 patient-years (6).

The distribution of infection-causing species significantly differs between NJI and prosthetic-joint infections (PJI). Septic native joint arthritis is usually monomicrobial, and typically, highly virulent pathogens are encountered (i.e., Staphylococcus aureus, beta-hemolytic streptococci, and Gram-negative rods) (2). As a consequence, infections usually present as acute disease, with prominent systemic and local signs of inflammation (7). PJI may be caused by a wide variety of different pathogens. These include organisms encountered in native joint arthritis but also low-virulence organism (e.g., coagulase-negative staphylococci [CoNS], *Cutibacterium* spp., viridans streptococci, and *Enterococcus* spp.), with a selective pathogenic potential associated with implanted devices (8).

Microbiological diagnosis is key to optimal patient management in joint infections and is usually based on using conventional culture techniques to grow and identify causative organisms from synovial fluid or intraoperative tissue specimens. Although culture is recognized as the gold standard, its sensitivity may be impaired, resulting in false-negative results. Specifically, long storage and transportation times may hamper bacterial growth, as will prior exposure to antimicrobials (8–10). In turn, culture-negative arthritis on the clinical side makes treatment with broad-spectrum pathogen coverage necessary, leading to unnecessary selective pressure and potential adverse events (10).

Given the obvious limitations associated with culture-based diagnostics, there is an evident need for accurate, culture-independent approaches to pathogen detection. Studies using panbacterial (and panfungal) approaches targeting the 16S/18S ribosomal RNA (rRNA) genes (sRNA) (Molzym, Germany), showed an additional benefit, if the culture remained negative. Nonetheless, the authors noted a concurrent risk of false-negative PCRs, due to low sensitivity, so culturing remains imperative (11). To tackle the low-sensitivity issue, commercially available multiplex systems (i.e., Unyvero) or syndromic PCR panels can be employed, though their benefit for management is still to be determined (9, 12).

The recently released BioFire joint infection (JI) panel identifies 31 causative pathogens and additionally 8 clinically relevant genetic resistance markers, including *mecA* and -*C*, *vanA* and -*B*, carbapenemase-encoding genes (i.e., *bla*_KPC_, *bla*_IMP_, *bla*_NDM_, *bla*_OXA-48_-like, and *bla*_VIM_), and the most prevalent extended-spectrum-β-lactamase (ESBL)-encoding gene (*bla*_CTX-M_) (13). Here, we evaluated the technical performance of the BioFire JI assay in a tertiary-care hospital in Germany by retrospectively comparing the results from the multiplex panel for 123 synovial fluid samples from patients with suspected joint infection against our current culture-based standard of care (SOC) and identification via matrix-assisted laser desorption ionization–time-of-flight (MALDI-TOF) mass spectrometry. Furthermore, we set out to elucidate the possible effect on patient management by chart review.

## MATERIALS AND METHODS

### Study setting and inclusion criteria.

**FIG 1:**
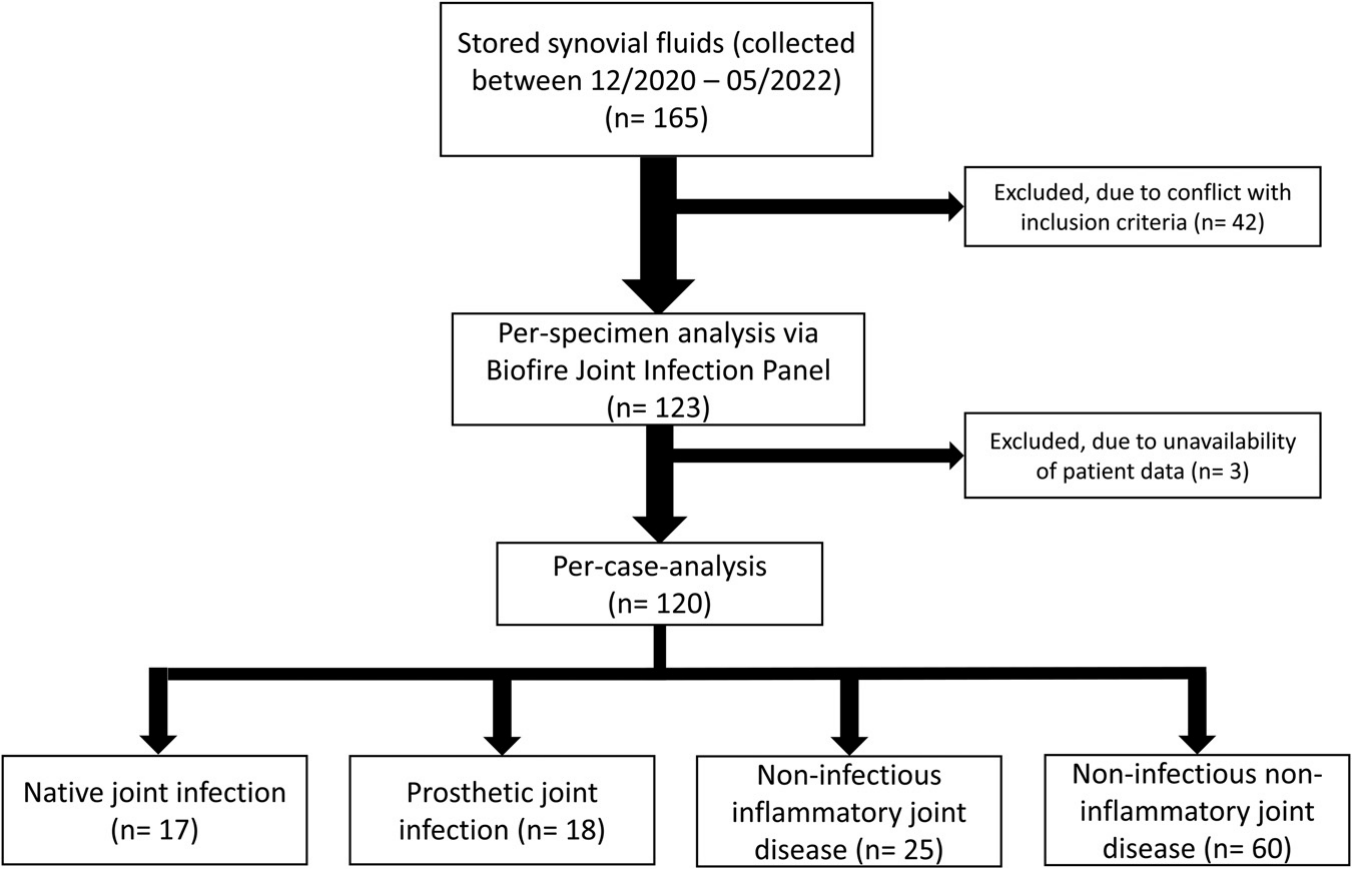
Flowchart of samples collected and included in the per-specimen- and per-case-analyses. The final diagnoses, retrieved from patients’ records, are also presented.

This retrospective single-center study was conducted at the University Medical Center Hamburg-Eppendorf, Germany, a 1,700-bed tertiary-care university hospital. All synovial fluid samples, which were analyzed according to routine microbiological workflows (described below) from December 2020 until May 2022, were stored at −20°C to −80°C and were eligible for analysis with the BioFire JI assay (BJA). Exclusion criteria for retrospective analysis using the BJA were follow-up arthrocentesis of the same joint within 30 days and a patient age of <18 years. Exclusion criteria for the per-case analysis were the above-mentioned criteria and unavailability of patient data. In total, 165 specimens were collected. After exclusion of 42 samples (age < 18, *n* = 2; follow-up arthrocentesis, *n* = 40), 123 samples from 123 patient cases ultimately underwent BJA testing. One hundred ten were tested with the research-use-only kit, whereas 13 were analyzed with the *in vitro* diagnostic (IVD) certified kit. Data from 120 cases were analyzed retrospectively for possible impact of BJA analysis on clinical patient management (Fig. 1). Three patients were excluded from per-case analysis due to unavailability of clinical data. Of note, three patients had synovial specimens obtained from two different joints, and therefore each joint is regarded as a case by itself.

### Conventional synovial fluid analysis (SOC).

Fifty microliters of synovial fluid was subjected to Gram staining via a PolyStainer (IUL Instruments). Fifty microliters of synovial fluid was spread onto Columbia sheep blood agar, chocolate agar, and Sabouraud agar for the detection of aerobic bacteria and yeasts and on Schaedler anaerobic agar (all from Oxoid, Basingstoke, UK) for cultivation of anaerobic bacteria. Incubation was carried out at 37°C and 5% CO_2_ for up to 14 days or under anaerobic conditions for 48 h. Plates were evaluated for growth after 24 h, 48 h, 7 days, and 14 days. In addition to solid media, 2 mL of thioglycolate broth was inoculated with up to 500 μL of the synovial fluid. All microorganisms obtained from cultures were subjected to pathogen identification using MALDI-TOF (Microflex; Bruker Daltonics, Bremen, Germany).

Isolates were subjected to susceptibility testing on a Vitek2 instrument (bioMérieux, Marcy l’Étoile, France) using either the Vitek2 AST-N223 (*Enterobacterales*) or the Vitek AST-P611 card (staphylococci and enterococci). Agar diffusion was employed to test fastidious organisms (e.g., streptococci) according to EUCAST protocols. Additionally, anaerobic bacteria were tested using brucella agar, McFarland 1.0, and anaerobic culturing conditions. Oxacillin resistance in S. aureus was confirmed using an immunochromatographic assay (Abbott, Scarborough, ME, USA).

### BioFire JI analysis.

BJA analysis was performed according to the manufacturer’s guidelines. Briefly, manufacturer-provided hydration solution was loaded into the pouch, and 200 μL of the synovial fluid was mixed with the provided sample buffer. This mixture was added to the pouch, which then was loaded into the instrument. Nucleic acid extraction, a multiplexed nested PCR, and a product melting temperature analysis were performed by the BioFire instrument. An overview of genera or families of bacteria and resistance markers represented in the version of the BJA used here is shown in Table S1 in the supplemental material.

### Verification of discrepant results.

To verify potentially discrepant results between culture and BJA analysis, respective specimens were subjected to a BJA rerun. If discrepant results were confirmed on rerun, PCRs specific for the species in question were employed. To this end, nucleic acids were extracted from the specimens using a MagNa Pure 96 system (Roche, Mannheim, Germany), and PCR was performed on a LightCycler 480 II instrument (Roche, Mannheim, Germany). Primers and probes were used as described previously (S. aureus [14], Streptococcus pneumoniae [15], and Bacteroides fragilis [16]).

### Clinical impact evaluation.

The primary objective of retrospectively analyzing patient and treatment characteristics was to identify the possibility for treatment improvement. Data were analyzed with regard to whether patients received optimal, adequate, or inadequate treatment. Optimal treatment was defined as the use of an antimicrobial substance with the narrowest possible spectrum of activity, taking into account indications and contraindications (according to local and international recommendations for the treatment of infectious diseases). Adequate antimicrobial therapy was defined as the use of an antimicrobial for which *in vitro* activity against the infecting microorganism was proven, while inadequate therapy was defined as therapy that did not cover the identified organism. De-escalation was defined as a change from a broad-spectrum antimicrobial therapy to an antibiotic with less broad-spectrum coverage, whereas escalation was defined as a change to a broad-spectrum antimicrobial therapy from an antibiotic with less broad-spectrum coverage.

The final diagnosis as NJI, PJI, inflammatory noninfectious joint disease (i.e., gout), or noninfectious noninflammatory joint disease was extracted from the final clinician’s report and used here for classification. PJI and NJI were diagnosed clinically (i.e., presentation of the joint, increased C-reactive protein (CRP), and increased leukocyte count in joint aspirate, if findings were available from the pathology department). The following data were additionally recorded by chart review, if available: demographics, joint type (native or prosthetic), synovial fluid analysis (cell count and neutrophil percentage), level of C-reactive protein in serum, histopathologic findings, and duration of the antimicrobial treatment administered before and after result of the SOC (empirical and targeted treatment).

### Quality control.

MALDI-TOF quality control (QC) was performed on a daily basis with Escherichia coli ATCC 25922. The EUCAST and Vitek2 quality control procedure was performed regularly once per week, as previously described (17). Full process control for the species-specific PCRs was performed by an internal spike-in control, which was added during DNA extraction (Cobas omni optimization reagent; Roche).

Additionally, the JI assay QC was performed once with the supplied positive and negative QC test vials.

### Statistics.

Sensitivity and specificity (including 95% confidence intervals [CI]) were calculated using the MedCalc webpage (https://www.medcalc.org/calc/diagnostic_test.php). All further basic statistical calculations were performed in Excel (Microsoft Office Professional Plus 2013).

### Ethics.

According to the Ethics Committee of the Hamburg Chamber of Physicians, no informed consent was required for the collection, analysis, and publication of these data (WF-045/21).

## RESULTS

### Per-specimen analysis.

To compare the diagnostic sensitivity of the BJA to that of SOC procedures, 123 synovial fluid samples obtained from 120 patients (one specimen/patient, *n* = 117; two specimens [independent joints]/patient, *n* = 3 patients) were analyzed. Microscopy identified Gram-positive cocci in 4/123 (3.3%) cases; all other specimens were negative for bacteria. The SOC identified bacterial pathogens in 15/123 (12.2%) synovial fluids, including 2/4 specimens that were Gram stain positive (Table 1). All isolates were cultivated directly on solid agar media without broth enrichment cultures. The BJA correctly identified all on-panel pathogens (9/15). Six specimens growing off-panel organisms (Staphylococcus epidermidis, *n* = 4; Cutibacterium acnes, *n* = 1; Micrococcus luteus, *n* = 1) were called negative by the BJA. The sensitivity and specificity of the BJA were 100% (95% CI, 69.2% to 100%) and 100% (95% CI, 96.8% to 100%), respectively, for on-panel pathogens. The overall BJA pathogen coverage rate in the collection under investigation was 60.0%. No resistance markers were detected by the BJA, which was in concordance with the antimicrobial susceptibility testing (AST) results obtained with the SOC.

**TABLE 1:** Overview of previously reported findings

Case no.	Joint type	Classification^*a*^	Result of:			CRP concn in serum (mg/L)	Synovial cell count (/μL)	Relevance of SOC findings^*b*^
			Gram staining	SOC	BJA^*c*^			
1	Prosthetic	PJI	Negative	Staphylococcus epidermidis	None	124	15,020	Yes
5	Native	NJI	Negative	Staphylococcus aureus	Staphylococcus aureus	228	No information	Yes
9	Native	NJI	Negative	Staphylococcus lugdunensis	Staphylococcus lugdunensis	99	42,440	Yes
10	Native	NJI	Negative	Staphylococcus epidermidis	None	39	37,320	Yes
14	Prosthetic	Noninflammatory, noninfectious	Negative	Staphylococcus epidermidis	None	37	No information	No
15	Prosthetic	PJI	Negative	Enterococcus faecalis	Enterococcus faecalis	71	58,040	Yes
29	Prosthetic	PJI	Negative	Staphylococcus epidermidis	None	104	26,660	Yes
32	Prosthetic	PJI	Negative	Cutibacterium acnes	None	8	11,660	Yes
35	Native	NJI	Gram-positive cocci	Streptococcus pyogenes	Streptococcus pyogenes	187	81,600	Yes
38	Native	NJI	Negative	Sterile	Bacteroides fragilis	258	86,200	NA
43	Native	NJI^*a*^	Negative	Micrococcus luteus	None	21	460	No^*d*^
69	Native	NJI	Negative	Sterile	Bacteroides fragilis	90	1,460	NA
70	Prosthetic	PJI	Gram-positive cocci	Staphylococcus aureus	Staphylococcus aureus	327	356,600	Yes
80	Prosthetic	PJI	Negative	Sterile	Streptococcus pneumoniae	51	80,440	NA
84	Prosthetic	PJI	Negative	Escherichia coli	Escherichia coli	110	No information	Yes
103	Prosthetic	Noninflammatory, noninfectious	Negative	Sterile	Staphylococcus aureus	46	900	NA
104	Prosthetic	PJI	Negative	Candida albicans	Candida albicans	253	11,840	Yes
114	Native	NJI	Negative	Streptococcus pyogenes	Streptococcus pyogenes	244	No information	Yes
119	Native	NJI	Negative	Enterobacter cloacae complex	Enterobacter cloacae complex	224	No information	Yes

a According to the clinician’s report.

b Yes, SOC results were considered clinically relevant; no, findings were not considered clinically relevant; NA, not applicable.

c “None” indicates a negative result due to an off-panel target (see Table S1 for a detailed overview of BJA targets).

d S. aureus was identified in intraoperative samples from a different joint and subsequently treated with flucloxacillin.

Strikingly, although the SOC remained negative, the BJA identified bacterial pathogens in four cases (Bacteroides fragilis, *n* = 2; Streptococcus pneumoniae, *n* = 1; Staphylococcus aureus, *n* = 1) (Table 1). In all of those four samples, the Gram stain did not reveal any bacteria. Employing in-house species-specific PCR, BJA results were validated in all cases and thus can be regarded as true positives. Of note, S. aureus was subsequently independently recovered from intraoperative tissue specimens.

### Per-case analysis.

The increased speed and sensitivity of the BJA compared to SOC culture approaches support the hypothesis that introduction of the molecular assay could have important implications for clinical management of arthritis patients. To test this idea, BJA results were also analyzed on a per-patient basis and related to clinical aspects in 120 individual cases.

The mean patient age was 65.1 (range, 18 to 93 years; standard deviation [SD], 17.8; 95% CI, 61.9 to 68.3). Most of the specimens were from knees (*n* = 79), followed by hip, shoulder, ankle, and elbow joints (*n* = 25, *n* = 10, *n* = 4 and *n* = 2, respectively). Sixty-six synovial fluid samples (55.0%) were obtained from native joints, while 54 (45.0%) were obtained from prosthetic joints. Seven of 66 synovial fluid samples from native joints and 8/54 specimens from prosthetic joints showed bacterial growth. The diagnoses, as stated in the final medical report, were native-joint infection (*n* = 17), PJI (*n* = 18), noninfectious inflammatory joint disease (*n* = 25), and noninfectious noninflammatory joint disease (*n* = 60). For more details, see Table S2.

### Hypothetical clinical impact.

For samples that showed bacterial growth by SOC testing, the first result available was the Gram stain result, with a median time of 6.37 h (SD, 5.02 h) after sampling. The median time to species identification was 50.29 h (SD, 6.42 h), whereas the median time until AST was 100.56 h (SD, 03.10 h). The mean BJA time to result was 60 min. Thus, compared to the SOC, BJA would have resulted in faster provision of species identification and molecular susceptibility data, by 49 h and 99 h, respectively.

Retrospective chart review of patients in whose samples pathogens were detected by either the SOC or the BJA (*n* = 19) revealed that in 10/19 (52.6%) cases, antibiotic therapy was started after synovial fluid was obtained for microbiological workup. Six patients had already received antibiotics prior to specimen collection. In 2/6 of these cases, the pathogen was identified only via the BJA (cases 69 and 103) (Table 1). In 2/19 cases, no antibiotic therapy was administered throughout the hospital stay. Here, the microbiological findings were interpreted as reflecting contamination (cases 14 and 31) (Table 1).

In 9/19 cases, pathogens were detected by the SOC and the BJA. Review of the antibiotic treatment of those nine cases (Table 2) showed that in one case, empirical therapy was optimal. In six cases, an adequate therapy was empirically initiated. In this group, six de-escalations were initiated after SOC results became available after 48 to 72 h. In two cases, initial therapy was inadequate. In both cases, the therapy was switched to optimal antibiotics after results from culture and AST became available.

**TABLE 2:** Overview of empirical and targeted therapies in cases with SOC- and BJA-positive specimens

Case no.	Result of SOC and BJA	Diagnosis (according to final medical report)	Antibiotic therapy^*a*^		Empiric therapy	Adapted therapy^*b*^
			Before specimen collection	After specimen collection		
5	S. aureus	Native-joint infection	0	1	Amoxicillin-clavulanic acid	Flucloxacillin, clindamycin
9	S. lugdunensis	Native-joint infection	0	1	Amoxicillin-clavulanic acid	Flucloxacillin, clindamycin
15	E. faecalis	Prosthetic-joint infection	0	1	Amoxicillin-clavulanic acid	Ampicillin, ceftriaxone
35	S. pyogenes	Native-joint infection	0	1	Meropenem, clindamycin	Penicillin G, clindamycin
70	S. aureus	Prosthetic-joint infection	0	1	Piperacillin-tazobactam	Flucloxacillin, gentamicin, ceftazidime
84	E. coli	Native-joint infection	0	1	Piperacillin-tazobactam	NA
104	C. albicans	Prosthetic-joint infection	1	1	Amoxicillin-clavulanic acid	Meropenem, fluconazole^*c*^
114	S. pyogenes	Native-joint infection	1	1	Amoxicillin-clavulanic acid	Penicillin G, clindamycin
119	E. cloacae complex	Native-joint infection	0	1	Vancomycin	Piperacillin-tazobactam

a 0, no; 1, yes.

b Antibiotic treatment after SOC results became available. NA, not applicable.

c Due to clinical worsening of the patient, meropenem was empirically added together with fluconazole.

Retrospective chart review of four SOC-negative, BJA-positive cases (Table 3) revealed that one PJI case (case 80) in which S. pneumoniae was detected did not receive antibiotic treatment. A second PJI case (case 103), in which BJA detected methicillin-susceptible S. aureus, had already received piperacillin-tazobactam prior to admission and joint puncture, but therapy was discontinued after negative SOC diagnostic results became available. Of note, the patient was readmitted to the hospital presenting with a PJI, and S. aureus was subsequently identified by SOC analysis. The third case (case 69), in which B. fragilis was identified only by BJA, received antimicrobial therapy prior to joint fluid aspiration for intravascular device endocarditis caused by S. aureus (Table 3). The other case with a BJA-identified B. fragilis isolate (case 38) received adequate but not optimal therapy (i.e., meropenem and daptomycin) for a total of 68 days. Thus, the BJA could have confirmed the diagnosis of joint infection more quickly in these four cases, helping to avoid the use of broad-spectrum antibiotics, i.e., meropenem and daptomycin, or helping to determine the appropriate therapy.

**TABLE 3:** Overview of species identified only via the BioFire joint infection panel^*a*^

Case no.	Result of:		Diagnosis^*b*^	Antimicrobial therapy		Adapted antimicrobial therapy^*c*^
	SOC	BJA		Before sample acquisition	After sample acquisition	
38	Sterile	B. fragilis	Native-joint infection	None	Meropenem	Meropenem, daptomycin
69	Sterile	B. fragilis	Native-joint infection	Flucloxacillin, ceftriaxone, fosfomycin, vancomycin, rifampicin	Cefazolin, rifampicin	None
80	Sterile	S. pneumoniae	Prosthetic-joint infection	None	None	None
103	Sterile	S. aureus	Noninfectious, noninflammatory joint disease	Piperacillin-tazobactam^*d*^	None	None

a The standard of care revealed no growth of bacterial pathogens.

b According to the final medical report.

c Antibiotic treatment after SOC results became available.

d Administered previously in a different hospital.

## DISCUSSION

Pathogen identification is of paramount importance for optimal patient management in bacterial arthritis. Current approaches include microscopy and culture, which is slow and can be difficult if patients have been pretreated with antibiotics or if difficult-to-culture pathogens are present. The BJA, allowing for rapid pathogen detection, has the potential to improve patient management in acute joint infections.

Here, the BJA was evaluated in a collection of 123 synovial fluid samples from patients presenting with symptoms consistent with acute bacterial joint infection or warranting an exclusion of this disease. Notably, only four specimens were positive by Gram staining, of which two were unambiguously validated by positive cultures and molecular pathogen detection. Negative cultures as well as a negative BJA suggest false-positive microscopy results in the remaining two, overall indicating the very limited usefulness of Gram staining in synovial fluid analysis. Fifteen of 123 specimens were culture positive, and the BJA correctly identified all on-panel pathogens (9/15). High diagnostic sensitivity to detect pathogens relevant in acute native-joint infections was also reported by recently published studies on the technical performance of the BJA (18–20), together with evidence from our study underpinning the good technical performance of the BJA in this clinical context.

Prosthetic-joint infections represent an independent but increasingly important subset of joint infections. Unsatisfyingly high numbers of culture-negative PJI have propelled interest in culture-independent approaches (21). Amplification assays targeting the 16S rRNA gene have been investigated for their use in PJI diagnostics; however, they appeared to be limited in terms of diagnostic sensitivity (9). Species-specific PCR provides a higher diagnostic sensitivity, and syndromic panel PCR assays have shown to provide clinically useful information in PJI management (22). Importantly, in addition to major pathogens relevant in native-joint infections, PJI may be caused by a significant number of additional pathogens, most importantly CoNS and *Cutibacterium* spp. (8). CoNS and Cutibacterium acnes are not represented on the BJA, making the assay less useful in a PJI setting. In fact, BJA evaluation in synovial fluid specimens obtained from PJI showed a low diagnostic sensitivity in early acute PJI, i.e., a clinical context in which CoNS play an important role (18, 20). The authors of those studies suggest a role for the BJA in a late acute (hematogenous) PJI setting, i.e., infections predominantly caused by organisms represented on the panel of the BJA.

Intriguingly, BJA detected pathogens in four cases, which were negative using SOC diagnostics. The prevalence of culture-negative native-joint infections has been described in other studies as being as high as 19%, which correlates with our findings here (21.1%) (23). The reasons for negative cultures are not well understood; however, administration of antibiotics before specimen collection and the presence of difficult-to-culture organisms are possible explanations (10, 24). Three of four specimens in which the causative pathogen was identified only with the BJA contained fastidious organisms (i.e., B. fragilis and S. pneumoniae), indicating that the BJA potentially has a higher sensitivity for a selected group of joint infection-related pathogens. Similar observations were also made by others (19), and in order to reduce the number of culture-negative cases, the BJA could have general importance in septic arthritis diagnostic algorithms. Retrospective chart review of culture-negative, BJA-positive cases indicated that the availability of pathogen diagnosis would have improved patient management in terms of anti-infective therapies. Additionally, chart review revealed that use of the BJA could enable timely optimization of antibiotic therapies, limiting unnecessary usage of broad-spectrum antibiotics. A recent retrospective analysis showed that in septic arthritis, delayed administration of appropriate therapy was associated with prolonged antibiotic therapy, prolonged length of hospital stay, and higher hospital costs (25). Thus, the potential importance for antimicrobial stewardship programs may support a broader use of the BJA as a frontline assay performed immediately after synovial fluid arrival in the lab. Future studies need to address the question of whether BJA may also help to exclude an infectious cause, supporting early discontinuation of empirical antibiotic treatment.

Implementation of molecular testing in standard microbiology workflows is potentially associated with difficulties (26). These include inappropriate test order strategies (e.g., in cases with weak suspicion for septic arthritis), incorrect interpretation of assay results due to lack of knowledge of test performance characteristics (i.e., sensitivity, specificity, positive predictive value [PPV], and negative predictive value [NPV]), and additional costs. Therefore, as is evident for other syndromic testing strategies using multiplex molecular assays, introduction of the BJA demands strict structuring of the whole diagnostic process. To this end, dedicated antimicrobial stewardship programs for bone and joint infection are of great value (27–30).

Our study has limitations related to the retrospective, monocentric study design. Furthermore, some patient data were not available or remained unclear. Final diagnoses of PJI/NJI were extracted from the clinicians’ reports, which also might be erroneous or a hypothesis, especially if no causative pathogen could be identified. A further limitation is the short incubation time of 48 h for anaerobic bacteria, which might have resulted in too low a sensitivity to detect these organisms. Another limitation is the small number of positive samples and the lack of resistant bacteria in our specimen collection. Therefore, no general conclusions can be drawn about the performance of the BJA across all species and resistance markers included in the panel. Further studies are warranted to analyze the performance of the panel and its impact on patient management and outcomes in a prospective study setting.

In conclusion, the BJA proved to be a powerful tool, especially in acute native-joint infections, accelerating pathogen detection and improving the sensitivity of diagnostic procedures. Caution is warranted if the BJA is intended for use in PJI, taking into account the inability to detect major causative pathogens (i.e., CoNS and *C. acnes*).
