# Supplementary material for: Performance and Hypothetical Impact on Joint Infection Management of the BioFire Joint Infection Panel: a Retrospective Analysis
Source: J Clin Microbiol. 2023 Jul 13;61(8):e00592-23. doi: 10.1128/jcm.00592-23 (PMC10446873; doi:10.1128/jcm.00592-23)
Supplement: Supplemental file 1 — Tables S1 and S2. Download jcm.00592-23-s0001.docx, DOCX file, 0.02 MB [file jcm.00592-23-s0001.docx]

**Supplementary Table 1: Species and resistance genes detected by the Biofire Joint Infection Panel**

| **GRAM-POSITIVE BACTERIA** | **GRAM-NEGATIVE BACTERIA** | **YEAST:** | **ANTIMICROBIAL RESISTANCE GENES:** |
| --- | --- | --- | --- |
| *Anaerococcus prevotii/vaginalis* |  |  |  |
| *Clostridium perfringens* | *Bacteroides fragilis* | *Candida*spp. | **Carbapenemases** |
| *Cutibacterium avidum/granulosum* | *Citrobacter* | *Candida albicans* | IMP |
| *Enterococcus faecalis* | *Enterobacter cloacae* complex |  | KPC |
| *Enterococcus faecium* | *Escherichia coli* |  | NDM |
| *Finegoldia magna* | *Haemophilus influenzae* |  | Oxa-48-like |
| *Parvimonas micra* | *Kingella kingae* |  | VIM |
| *Peptoniphilus* | *Klebsiella aerogenes* |  | **ESBL** |
| *Peptostreptococcus anaerobius* | *Klebsiella pneumoniae* group |  | CTX-M |
| *Staphylococcus aureus* | *Morganella morganii* |  | **Methicillin Resistance** |
| *Staphylococcus lugdunensis* | *Neisseria gonorrhoeae* |  | *mecA/C* and MREJ (MRSA) |
| *Streptococcus* spp*.* | *Proteus* spp*.* |  | **Vancomycin Resistance** |
| *Streptococcus agalactiae* | *Pseudomonas aeruginosa* |  | *vanA/B* |
| *Streptococcus pneumoniae* | *Salmonella* spp*.* |  |  |
| *Streptococcus pyogenes* | *Serratia marcescens* |  |  |

**Supplementary Table 2: Patient characteristics**

|  | Patients analyzed by chart review (n= 120) |
| --- | --- |
| ***Baseline characteristics*** |  |
| Sex, female (%) | 44 (36.7 %) |
| Age, years, mean (SD; 95 % CI) | 65.1 (17,8; 61.9-68.3) |
| Prosthetic joint present | 54 (45.0 %) |
| ***Final diagnosis*** |  |
| Native joint infection (%) | 17 (14.2 %) |
| Prostehtic joint infection (%) | 18 (15.0 %) |
| non-infectious, inflammatory joint disease (%) | 25 (20.8 %) |
| non-infectious, non-inflammatory joint disease (%) | 60 (50.0 %) |
| ***Arthocentesis from following joint:*** |  |
| Knee (%) | 79 (65.8 %) |
| Hip (%) | 25 (20.8 %) |
| Shoulder (%) | 10 (8.3 %) |
| Ankle (%) | 4 (3.3 %) |
| Elbow (%) | 2 (1.7 %) |
